# Research on a machine learning-based predictive model for postoperative neurological dysfunction in acute Stanford type A aortic dissection

**DOI:** 10.3389/fmed.2026.1716649

**Published:** 2026-02-13

**Authors:** Lun Li, Ruiyi Wang, Lei Qin, Xiaoyong Jing, Junming Zhu

**Affiliations:** 1 Department of Cardiac Surgery, Beijing Anzhen Hospital, Capital Medical University, Beijing, China; 2 Beijing Lab for Cardiovascular Precision Medicine, Beijing, China; 3 School of Statistics, University of International Business and Economics, Beijing, China; 4 Dong Fureng Institute of Economic and Social Development, Dong Fureng Institute of Economic and Social Development, Wuhan University, Wuhan, China

**Keywords:** acute Stanford type A aortic dissection, machine learning, neurological dysfunction, prediction model, XGBoost model

## Abstract

**Introduction:**

This study aimed to construct and validate a machine learning (ML) model integrating preoperative, intraoperative, and postoperative multimodal clinical data for individualized prediction of postoperative neurological dysfunction (ND) in patients with acute Stanford type A aortic dissection (ATAAD).

**Methods:**

A retrospective analysis was conducted on 1,228 ATAAD patients (Aortic Disease Center of Beijing Anzhen Hospital, January 2020–December 2023): 853 patients (January 2020–December 2022) for model training/internal validation (via 10-fold cross-validation) and 375 patients (January–December 2023) for external validation. The 853 patients were grouped into control (*n* = 616) and ND (*n* = 237), including 203 transient ND (TND) and 34 permanent ND (PND) groups. Data were analyzed using Mann–Whitney *U*, chi-square (*χ*^2^), and Fisher’s exact tests (*p* < 0.05). Four ML models (SVC-LK, Nu-SVC, AdaBoost, XGBoost) were built with perioperative data; SHapley Additive exPlanations (SHAP) selected 15 robust features from 49 initial ones. Model performance was assessed via ROC-AUC (10-fold cross-validation for training/internal validation, external validation for effectiveness), and the optimal model was identified using DeLong test (two-tailed *p*-values). A multidimensional analysis compared the optimal model with traditional logistic regression (LR).

**Results:**

The XGBoost model exhibited the best performance: AUC = 0.966 (internal validation) and AUC = 0.951 (external validation), outperforming LR and the other three ML models.

**Conclusion:**

The XGBoost algorithm demonstrates superior efficacy in predicting postoperative ND in acute ATAAD patients, providing postoperative early warning, identifying high-risk patients, offering clinical guidance, and enabling timely intervention.

## 1 Introduction

Acute Stanford type A aortic dissection (ATAAD) is a life-threatening cardiovascular emergency with an extremely high mortality rate—1–2% per hour without surgical intervention, reaching 50% within 24 h and 90% within two weeks (1, 2). Although the “Sun’s Procedure” (total aortic arch replacement with stented elephant trunk implantation) has reduced surgical mortality from 30 to 5–7.5% in China over two decades (3), ATAAD surgery remains technically demanding. It involves complex procedures such as deep hypothermic circulatory arrest and selective antegrade cerebral perfusion (SACP), with an operation time exceeding 5–6 h even in high-level centers, and is accompanied by multiple intraoperative physiological disturbances (4).

Neurological dysfunction (ND) is one of the most common and destructive complications after ATAAD surgery affecting the long-term quality of life of patients (5). Clinical data show that the overall incidence of postoperative complications of the central nervous system is 37.5%. Postoperative ND is divided into two types according to the duration and severity of nerve injury:

1. Transient ND (TND): characterized by postoperative delirium, confusion, or transient neurological deficits, with an incidence rate ranging from 18 to 37.5%. Although most of these dysfunctions are reversible, they significantly prolong the duration of mechanical ventilation and increase hospitalization costs.
2. Permanent ND (PND): characterized by cerebral infarction, irreversible coma, or paraplegia caused by spinal cord injury. Although its incidence rate is relatively low (2.9–5%), PND leads to catastrophic consequences (6). Its occurrence significantly prolongs the length of stay in the ICU, the overall hospital stay and doubles the 30-day mortality rate of patients.

Among the two types, PND has attracted special attention due to its irreversibility (7).

Despite the significant clinical impact of postoperative ND, its accurate prediction and early intervention are still challenging, and existing risk prediction tools have notable limitations.

Machine learning (ML) offers promising solutions for improving ND prediction, but the selection of appropriate algorithms must align with clinical data characteristics. Support vector classification (SVC) with linear kernel (LK) is suitable for linearly separable clinical datasets, ensuring maximum classification margin while enhancing generalization (8). Nu-SVC (with radial basis function kernel) addresses non-linear relationships and complex feature interactions often present in ATAAD patient data (9). Adaptive boosting (AdaBoost) optimizes performance by focusing on hard-to-classify samples, making it effective for noisy or imbalanced clinical datasets-such as capturing interactions between deep hypothermic circulatory arrest duration and blood lactate levels (10). eXtreme Gradient Boosting (XGBoost) efficiently processes high-dimensional data, handles missing values common in retrospective clinical studies, and selects core predictive factors, which is critical for refining ND risk indicators (11).

Current postoperative ND prediction for ATAAD patients relies on traditional tools [e.g., logistic regression (LR)] with insufficient accuracy, and few studies have systematically applied ML algorithms tailored to clinical data characteristics for this specific complication. Through different modeling approaches, the four algorithm types handle linear/non-linear features and complex data patterns, serving as varied tools for constructing ML prediction models.

The focus of this study is to investigate whether machine learning-based models (SVC, Nu-SVC, AdaBoost and XGBoost) can achieve superior performance in predicting postoperative ND in patients with ATAAD compared to traditional LR models and existing scoring systems. It fills the gap in accurate ND prediction for ATAAD patients by leveraging ML algorithms optimized for clinical data features. It also provides a more reliable tool for early identification of high-risk patients, facilitating timely intervention to reduce ND-related morbidity and mortality.

## 2 Methods

### 2.1 Data collection

#### 2.1.1 Subjects

Data for this study were collected from the electronic medical record of patients who underwent surgery for ATAAD at the Pediatric Cardiac Center of Beijing Anzhen Hospital, Capital Medical University, from 2020 to 2023. A total of 1,228 patients were included, and 49 variables encompassing the entire perioperative process were collected, including:

1. Baseline characteristics: demographic information, living habits, underlying diseases, and preoperative organ function status.
2. Anatomical characteristics of the dissection and surgical details: cerebral perfusion method, circulatory arrest time, and aortic arch reconstruction approach.
3. Postoperative recovery indicators: duration of mechanical ventilation, length of ICU stay, and complications such as low cardiac output and renal insufficiency.
4. Core outcome indicator: postoperative ND and neurological status during the follow-up period.

The technique of moderate hypothermic circulatory arrest (MHCA) combined with SACP was uniformly used for combined aortic arch surgery.

#### 2.1.2 Surgical details

All patients were placed in a supine position, and a median sternotomy was performed to open the chest. Anesthesia was maintained by total intravenous anesthesia (propofol combined with sufentanil) together with inhalational anesthesia (sevoflurane), supplemented with vecuronium. Tranexamic acid was administered to support the coagulation function. Arterial cannulation was performed after systemic heparinization through the right axillary artery, femoral artery, and/or ascending aorta; venous cannulation was performed through the right atrium to establish cardiopulmonary bypass. Cooling was carried out under parallel circulation, and left heart drainage was placed through the right upper pulmonary vein, with the flow rate maintained at 2.2–2.5 L/min/m^2^. The myocardium was protected by perfusing cold cardioplegic solution through the left and right coronary arteries.

Arterial cannulation was performed on the ascending aorta when the lesion was confined to this region, while venous cannulation was consistently established through the right atrium, followed by the reconstruction of the ascending aorta. The patient’s body temperature was lowered to 28 °C, the brachiocephalic vessels were clamped, and unilateral or bilateral antegrade selective cerebral perfusion was applied under moderate hypothermic circulatory arrest (26–28 °C) when the lesion involved the aortic arch and the reconstruction of the distal aorta or aortic arch was necessary. An intraoperative stent was placed in the descending aorta distal to the left subclavian artery. The distal end of the four-branched artificial blood vessel was anastomosed to the proximal end of the descending aortic stent using a single continuously 4/0 monofilament suture. Cardiopulmonary bypass perfusion was resumed through the fourth branch, and the proximal end of the four-branched artificial blood vessel was anastomosed to the ascending aortic artificial blood vessel. The heart was defibrillated, and beating was resumed after rewarming and air evacuation. Subsequently, the left common carotid artery, left subclavian artery, and innominate artery were sequentially anastomosed using continuous 5/0 monofilament suture.

Concurrent cardiac surgical procedures were performed during the core cooling phase depending on the patient’s condition. The surgical strategies for the aortic root included the Bentall procedure, Wheat procedure, and David procedure while those for the aortic arch included partial aortic arch replacement, aortic arch island anastomosis, and total aortic arch replacement.

### 2.2 Data analysis

#### 2.2.1 Data preprocessing

The core goal of data preprocessing in this study was to ensure data quality and provide reliable input for the subsequent construction of ML models. All clinical indicators included in the study were complete due to the well-established research database. Therefore, no missing value imputation or sample exclusion was required, and the original information of all patients was fully preserved, ensuring the integrity and authenticity of the data.

On this basis, data were further standardized by performing distribution characteristic tests and outlier identification for continuous variables (such as age, weight, and various laboratory test indicators). Conversion for categorical variables (such as sex, medical history type, and surgical method), was carried out to meet the format requirements of the input data of ML algorithms. Data loss was effectively reduced since the entire preprocessing procedure was not affected by missing values, providing a reliable data basis for subsequent model training and validation.

#### 2.2.2 Statistical analyses

Descriptive data analysis and basic statistical analysis were performed on 1,228 subjects in the database. Continuous variables were expressed as median and interquartile range, while categorical variables were expressed as frequency and percentage. Statistical analysis of continuous variables was performed using the Mann–Whitney *U* test, while that of categorical variables was performed using the chi-square test and Fisher’s exact test. The area under the receiver operating characteristic (ROC) curve and area under the curve (AUC) were compared among ML prediction models. Two-tailed *p*-values were calculated using the DeLong test, with a *p* < 0.05 considered statistically significant.

### 2.3 Model specification

#### 2.3.1 Feature selection

All demographic features and preoperative clinical data were included as features in the ML models to improve the accuracy of the prediction. This selection was intended to reflect both individual differences and baseline disease characteristics, offering the model diverse input to uncover potential predictive patterns.

A total of 49 features of the subjects were included at the initial phase of model training. The SHapley Additive exPlanations (SHAP) method was used to identify the features with a significant impact on prediction results. This method not only quantifies the overall importance of each feature in prediction but also provides an in-depth assessment of the direction and magnitude of each feature’s influence on individual patient predictions, thus offering a detailed and interpretable basis for evaluating the importance of features. Finally, the features identified as important in all prediction models were selected as the final inputs of the ML model. This selection process preserved essential information while minimizing the influence of redundant features on model performance.

The presence or absence of postoperative neurological abnormalities was used as the target variable during model training, and a total of 48 variables were used as input features, including basic information (such as age, sex, height, smoking history, and history of hypertension), preoperative indicators (such as preoperative renal dysfunction, preoperative hemoglobin, and preoperative platelets), intraoperative variables (such as arterial cannulation method, cerebral perfusion method, and operation time), and postoperative/follow-up variables (such as postoperative length of hospital stay, extreme value of postoperative hemoglobin, and reoperation).

#### 2.3.2 Construction of ML prediction models

Data of 1,228 subjects from the database were used for building ML prediction models. Among them, data from 853 subjects from January 2020 to December 2022 were used for model training and internal validation, while data from 375 subjects of January–December 2023 were used for external validation. A 10-fold cross-validation method was used to split the data for training and internal validation.

In the selection of ML classification algorithms, SVM-related models and ensemble learning methods are two commonly used and effective types of tools. Among them, the linear kernel of the support vector classifier (SVC) performs a classification by constructing an optimal linear hyperplane in the feature space. It is suitable for handling linearly separable datasets and effectively improves the model’s generalization ability while ensuring the maximization of the classification margin. As a variant of SVC, Nu-SVC uses a radial basis function kernel that maps non-linear data in the low-dimensional space to the high-dimensional feature space through non-linear mapping, enabling it to handle high-dimensional complex relationships and non-linear classification tasks. This makes it particularly useful for scenarios with complex interaction effects among features.

The AdaBoost algorithm is a type of ensemble learning method that constructs a strong classifier by iteratively integrating multiple base decision trees. Its core mechanism consists of adjusting the weight of misclassified samples, strengthening the learning of hard-to-classify samples, gradually optimizing the model performance. It shows good adaptability when dealing with noisy or imbalanced data and effectively captures complex interaction effects, such as those between the duration of deep hypothermic circulatory arrest and blood lactate levels.

The eXtreme Gradient Boosting (XGBoost) algorithm, on the other hand, is based on a gradient boosting framework. It detects model deficiencies using negative gradient information, iteratively refines the model by generating new decision trees to fit the negative gradient, and simultaneously incorporates a regularization mechanism to mitigate the risk of overfitting. XGBoost offers the advantage of efficiently managing high-dimensional data and complex feature interactions; it further addresses missing values commonly present in retrospective clinical studies and identifies core predictive factors among the 49-dimensional features of patients.

These four types of algorithms address the linear/non-linear features and complex patterns of data through different modeling logics, providing a variety of options for the construction of ML prediction models.

### 2.4 Assessment of models

#### 2.4.1 10-fold cross-validation

Since ML algorithms require a strict separation of training and testing data, a 10-fold cross-validation was used for model evaluation and parameter optimization, a widely recognized reliable strategy for improving model generalization ability. The implementation proceeded as follows: first, the internal validation cohort (853 cases) was equally divided into 10 subsets; 9 were used for model training, while the remaining subset was used as an independent test. This procedure was repeated 10 times ensuring that each subset was used once for testing, thereby completing a full cross-validation cycle. Finally, the mean of the 10 testing results was calculated to evaluate model performance (e.g., AUC).

Since several hyperparameters involved in the classification model significantly affected the model performance, the Grid Search algorithm was used to identify the optimal hyperparameter combination: first, the parameter search space for each classifier was predefined, such as parameters like the penalty coefficient for SVC and the kernel bandwidth for Nu-SVC. Next, 10-fold cross-validation was used to evaluate the performance of each parameter set, selecting the combination that achieved the highest cross-validation AUC. The optimal parameter combination for each algorithm was finally determined after multiple iterations refining the search ranges.

The comparison of classification algorithms was also performed based on 10-fold cross-validation. The ROC curve and AUC value were calculated during each validation process. The classifier algorithm with the best performance was finally selected by comparing the mean and standard deviation of AUC among different algorithms, providing a reliable basis to select the best algorithm in model construction.

#### 2.4.2 Model evaluation

This study used a multi-dimensional comparative analysis to comprehensively assess the performance of the constructed ML prediction models and clarify their advantages over traditional prediction models, as well as compare them with traditional prediction models.

As regards the traditional prediction model, a multivariate LR model with postoperative neurological function as the endpoint event was constructed using multivariate LR analysis. During the internal validation phase, a 10-fold cross-validation method was used to evaluate the ML prediction models. The ROC curve and AUC were calculated for each validation round, and the mean value of these AUCs was used as a quantitative indicator of the model’s internal discriminative ability, with a higher AUC value indicating a stronger ability of the model to distinguish between event occurrence and non-occurrence. The ROC curve and AUC of the traditional prediction model were calculated using the internal validation data, allowing for a preliminary comparison of performance between the ML models and the traditional model in the internal dataset.

External validation was performed to assess the generalization of the models, following this procedure: first, the ML prediction models were trained using the internal validation dataset. Next, a parameter calibration using the Platt logistic model was applied to adjust the predicted probabilities of the models, optimizing their alignment with the actual event probabilities. The Brier score was then calculated to evaluate both discriminations (the model’s ability to distinguish between events and non-events) and calibration (i.e., the accuracy of predicted probabilities), with lower Brier score indicating better predictive performance of the model. Finally, using the external validation dataset, the performance of the trained and calibrated ML models was comprehensively compared with that of the multivariate LR prediction model to verify the applicability and stability of the ML models in external populations.

The clinical application of the ML prediction models was objectively evaluated from multiple dimensions through the aforementioned internal and external validations, as well as multi-model comparisons, and included the discriminative ability, calibration effect, and generalization performance.

## 3 Results

### 3.1 Subject characteristics

The characteristics of the patients included in this work are listed in Table 1. The median age was 45 years (range: 0.51–60), and 318 were female (37.28%).

**Table 1:** Subject characteristics.

Characteristic	Overall (*n* = 853)	Without ND (*n* = 616)	Combined ND (*n* = 237)
Age (year)	45 (0.51–60)	45 (51–59)	41 (53–62)
Gender, female *n* (%)	318 (37.28%)	417 (67.69%)	118 (49.79%)
Weight (kg)	75 (65–81)	65 (75–80)	66 (70–92)
Height (m)	1.71 (1.62–1.77)	1.71 (1.63–1.75)	1.72 (1.61–1.80)
BMI (kg/m^2^)	26 (23–28)	23 (25–28)	24 (26–30)
Preoperative cardiac function, grade 3 *n* (%)	528 (61.90%)	406 (65.91%)	122 (51.48%)
Preoperative pericardial effusion *n* (%)	573 (67.17%)	390 (63.31%)	183 (77.22%)
Smoking history *n* (%)	355 (41.62%)	272 (44.16%)	83 (35.02%)
Hyperlipidemia *n* (%)	252 (29.54%)	184 (29.87%)	68 (28.69%)
History of coronary heart disease *n* (%)	103 (12.08%)	76 (12.34%)	27 (11.39%)
History of hypertension *n* (%)	641 (75.15%)	456 (74.03%)	185 (78.06%)
History of diabetes mellitus *n* (%)	218 (25.56%)	141 (22.89%)	77 (32.49%)
History of cerebrovascular disease *n* (%)	125 (14.65%)	60 (9.74%)	65 (27.43%)
Preoperative renal dysfunction *n* (%)	46 (5.39%)	12 (1.95%)	34 (14.35%)
Carotid artery stenosis or occlusion *n* (%)	188 (22.04%)	94 (15.26%)	94 (39.66%)
History of cardiac surgery *n* (%)	107 (12.54%)	64 (10.39%)	43 (18.14%)
Preoperative hypoxemia *n* (%)	53 (6.21%)	18 (2.92%)	35 (14.77%)
Preoperative hemoglobin (g/L)	133 (121–148)	132 (124–148)	134 (114–153)
Preoperative platelets (*10^9^/L)	179 (146–217)	189 (150–214)	174 (135–218)
Preoperative blood lactate (mmol/L)	1.8 (1.2–2.6)	1.8 (1.2–2.4)	1.6 (1.2–3)
Preoperative creatinine (μmol/L)	83 (66–105)	85 (68–105)	80 (59–97)
Dissection type, A2 *n* (%)	349 (40.91%)	253 (41.07%)	96 (40.51%)
Refined type, C *n* (%)	797 (93.43%)	560 (90.91%)	237 (100.00%)
Axillary arterial cannulation method *n* (%)	449 (52.64%)	341 (55.36%)	108 (45.57%)
Unilateral anterograde cerebral perfusion *n* (%)	579 (67.88%)	396 (64.29%)	183 (77.22%)
Coronary artery involvement *n* (%)	384 (45.02%)	261 (42.37%)	123 (51.90%)
Involvement of three branches of aortic arch *n* (%)	714 (83.70%)	496 (80.52%)	218 (91.98%)
Aortic root treatment method, ascending aortoplasty *n* (%)	448 (52.52%)	332 (53.90%)	116 (48.95%)
Aortic arch treatment method, total arch replacement plus elephant trunk stent surgery *n* (%)	480 (56.27%)	337 (54.71%)	143 (60.34%)
Operation time (hour)	7 (6–8)	6 (6–7)	7 (6–9)
Cardiopulmonary bypass time (min)	182 (144–218)	174 (140–200)	204 (159–233)
Aortic cross-clamp time (min)	98 (74–119)	84 (73–108)	113 (98–139)
Deep hypothermic circulatory arrest time (min)	22 (17–33)	22 (17–31)	23 (18–35)
Selective cerebral perfusion time (min)	22 (17–33)	22 (17–31)	23 (18–35)
Hospital stay (day)	15 (11–20)	15 (11–19.25)	15 (11–37)
ICU stay (hour)	72 (36–120)	68 (24–96)	96 (52–232)
Postoperative intubation time (hour)	31 (13–60)	27 (11–54)	50 (21–96)
Postoperative hospital stay (day)	12 (9–16)	10 (9–15)	14 (9–20)
Postoperative hypoxemia *n* (%)	402 (47.13%)	218 (35.39%)	184 (77.64%)
Postoperative hemoglobin extremum (g/L)	79 (68–88)	81 (72–92)	69 (61–81)
Postoperative platelet extremum (*10^9^/L)	81 (54–102)	84 (70–105)	59 (31–99)
Postoperative creatinine peak (μmol/L)	125 (91–221)	123 (91–209)	142 (89–304)
Postoperative renal insufficiency *n* (%)	376 (44.08%)	243 (39.45%)	133 (56.12%)
Postoperative low cardiac output syndrome *n* (%)	254 (29.78%)	108 (17.53%)	146 (61.60%)
Reoperation *n* (%)	35 (4.10%)	11 (1.79%)	24 (10.13%)
Abnormal neurological function during follow-up *n* (%)	215 (25.21%)	0 (0.00%)	237 (100.00%)
Follow-up time (month)	36 (30–42)	36 (30–42)	36 (24–42)
Death during follow-up *n* (%)	79 (9.26%)	0 (0.00%)	79 (33.33%)

### 3.2 Postoperative ND group

In this study, 237 subjects showed postoperative ND, accounting for 27.78% of the test set subjects. The clinical characteristics and prognosis of this group of patients were significantly different from those of the group without postoperative ND, and the specific manifestations were as follows: as regards preoperative comorbidities, the rate of patients with preoperative pericardial effusion in this group was 77.22%, which was significantly higher than that of the total population (67.17%) and the group without ND (63.31%). The incidence rates of cerebrovascular disease history (27.43%), preoperative renal insufficiency (14.35%), and carotid artery stenosis or occlusion (39.66%) were significantly higher than those of the other two groups, suggesting that the preoperative cerebrovascular and renal function status was related to postoperative ND. In addition, the rates of hypertension history (78.06%) and diabetes history (32.49%) in this group were also higher than those in the group without ND.

As regards surgery-related indicators, the operation time (7.29 h vs. 6.68 h), cardiopulmonary bypass time (204 min vs. 174 min), aortic cross-clamp time (113 min vs. 84 min), moderate hypothermic circulatory arrest time (23 min vs. 22 min), and selective cerebral perfusion time (23 min vs. 22 min) of patients in the postoperative ND group were longer than those in the group without ND, suggesting that surgical trauma and prolonged cerebral ischemia time increased the risk of ND.

As regards the prognosis, the postoperative recovery of this group of patients was significantly slower. The median length of ICU stay was 96 h, which was much longer than 68 h in the group without ND; the postoperative intubation time (50 h vs. 27 h) and postoperative hospital stay (14 days vs. 10 days) were also significantly prolonged. The incidence of postoperative complications increased significantly; the rates of postoperative hypoxemia (77.64%), postoperative renal insufficiency (56.12%), low cardiac output syndrome (61.6%), and reoperation (10.13%) were significantly higher than those in the group without ND. More importantly, the follow-up mortality rate of this group of patients was 33.33%, while that of the group without ND was 0, suggesting that postoperative ND was closely related to poor prognosis.

### 3.3 Risk factors for postoperative ND

This study used the multi-variable forward LR method to identify the independent risk factors related to postoperative ND, including clinically relevant features for analysis. The results are shown in Table 2. The analysis showed that sex, weight, height, history of cardiac surgery, history of cerebrovascular disease, smoking history, preoperative pericardial effusion, and preoperative renal insufficiency were independent risk factors affecting the occurrence of postoperative ND (all *p* < 0.05).

**Table 2:** Results of forward logistic regression.

Feature	Regression coefficient	Standard error	*p*-value	OR	OR 95% CI
Gender	0.897	0.144	0.000	2.452	1.850–3.250
Body weight	0.600	0.143	0.000	1.822	1.376–2.412
Height	0.255	0.120	0.034	1.290	1.020–1.633
History of cardiac surgery	0.889	0.261	0.001	2.432	1.459–4.054
History of cerebrovascular disease	1.458	0.262	0.000	4.297	2.573–7.176
Smoking history	−0.688	0.243	0.005	0.503	0.312–0.810
Preoperative pericardial effusion	1.035	0.209	0.000	2.816	1.870–4.241
Preoperative renal dysfunction	1.744	0.390	0.000	5.721	2.664–12.287
Intercept	−2.032	0.197	0.000	—	—

Specifically, sex (OR = 2.452, 95% CI: 1.850–3.250, *p* = 0.000), weight (OR = 1.822, 95% CI: 1.376–2.412, *p* = 0.000), and height (OR = 1.290, 95% CI: 1.020–1.633, *p* = 0.034) were risk factors for postoperative ND, suggesting that females and increased weight and height increased the risk of postoperative ND.

Among preoperative comorbidities, history of cardiac surgery (OR = 2.432, 95% CI: 1.459–4.054, *p* = 0.001), history of cerebrovascular disease (OR = 4.297, 95% CI: 2.573–7.176, *p* = 0.000), preoperative pericardial effusion (OR = 2.816, 95% CI: 1.870–4.241, *p* = 0.000), and preoperative renal insufficiency (OR = 5.721, 95% CI: 2.664–12.287, *p* = 0.000) were significant risk factors. Among them, preoperative renal insufficiency had the highest OR value, suggesting its closest association with postoperative ND. The history of cerebrovascular disease also showed a strong risk association, further confirming the important impact of preoperative cerebrovascular status on postoperative neurological function.

It is worth noting that smoking history was a protective factor for postoperative ND (OR = 0.503, 95% CI: 0.312–0.810, *p* = 0.005), suggesting that patients with smoking history had a lower risk of developing postoperative ND. However, this result needs to be further confirmed in combination with the clinical background.

### 3.4 ML prediction model

#### 3.4.1 Feature selection

**Figure 1:**
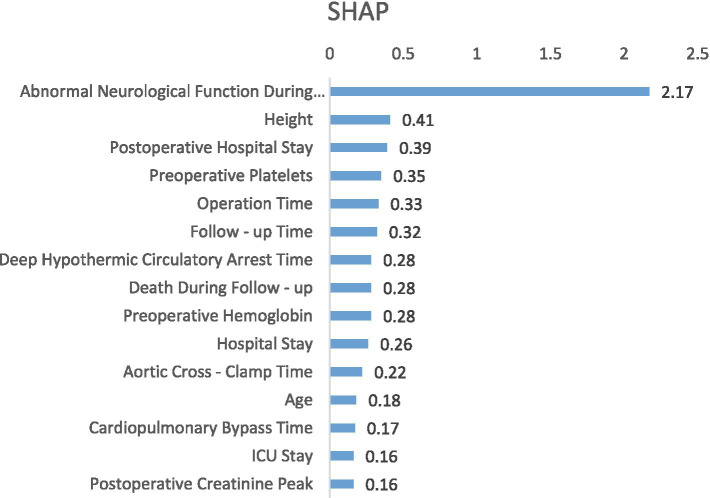
XGBoost feature importance ranking chart.

The ML model was constructed using preoperative features and unoptimized parameters, then the feature importance of the model was analyzed using the SHAP method. Finally, 15 features were selected to build the ML prediction model, as shown in Table 3. The ranking of feature importance for the XGBoost-based model is shown in Figure 1.

**Table 3:** Top 15 features selected by SHAP.

Top 15 features by importance
Abnormal neurological function during follow-up
Height (m)
Postoperative hospital stay (day)
Preoperative platelets (*10^9^/L)
Operation time (hour)
Follow-up time (month)
Deep hypothermic circulatory arrest time (min)
Death during follow-up
Preoperative hemoglobin (g/L)
Hospital stay (day)
Aortic cross-clamp time (min)
Age (year)
Cardiopulmonary bypass time (min)
ICU stay (hour)
Postoperative creatinine peak (μmol/L)

#### 3.4.2 Internal validation

**Figure 2:**
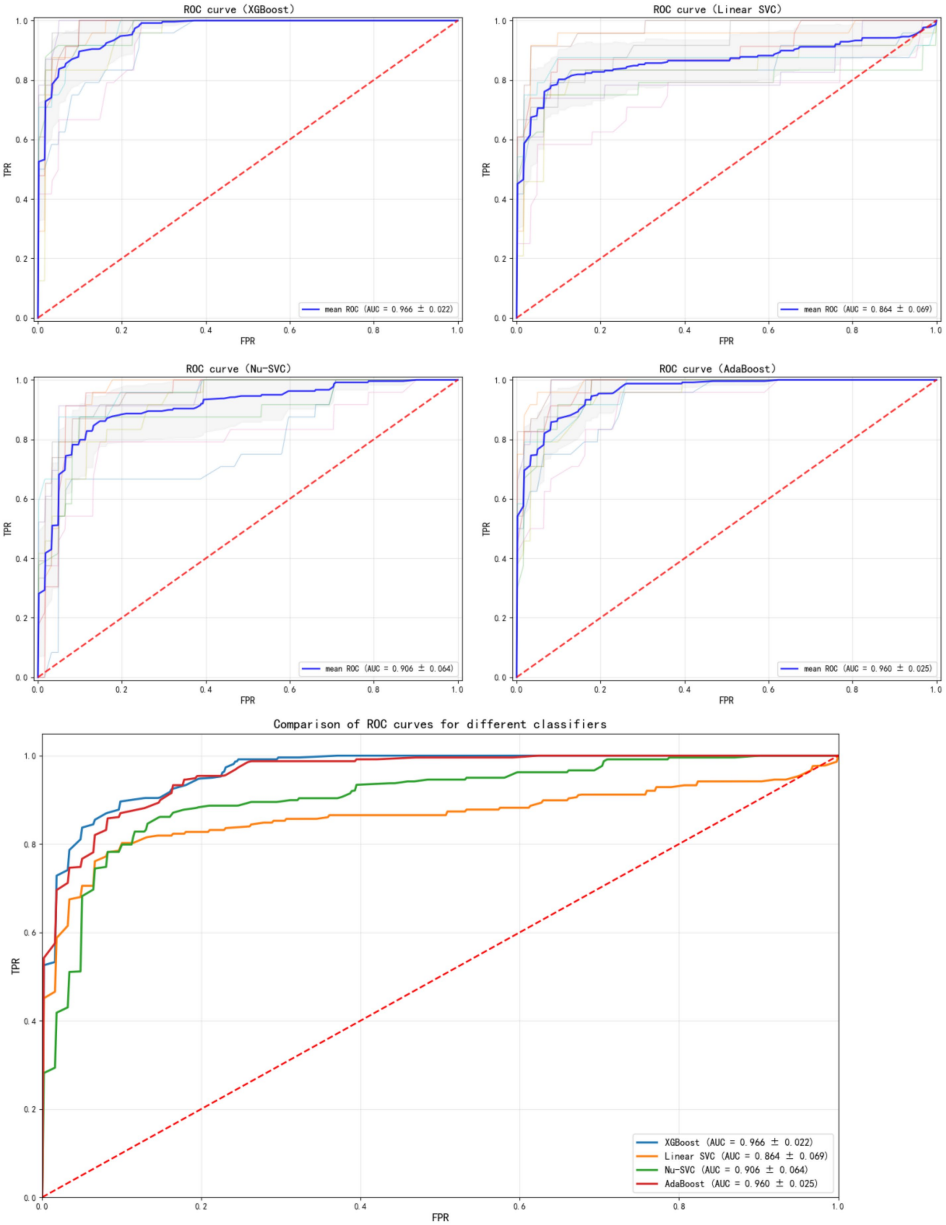
ROC curves of different models.

The results in Figure 2 show the ROC curves and AUC values of each ML model after 10-fold cross-validation. Among the prediction models, the XGBoost ML model showed the best performance (AUC = 0.966, 95% CI: 0.943–0.989). Therefore, this model was selected as the final ML model to evaluate its performance.

**Figure 3:**
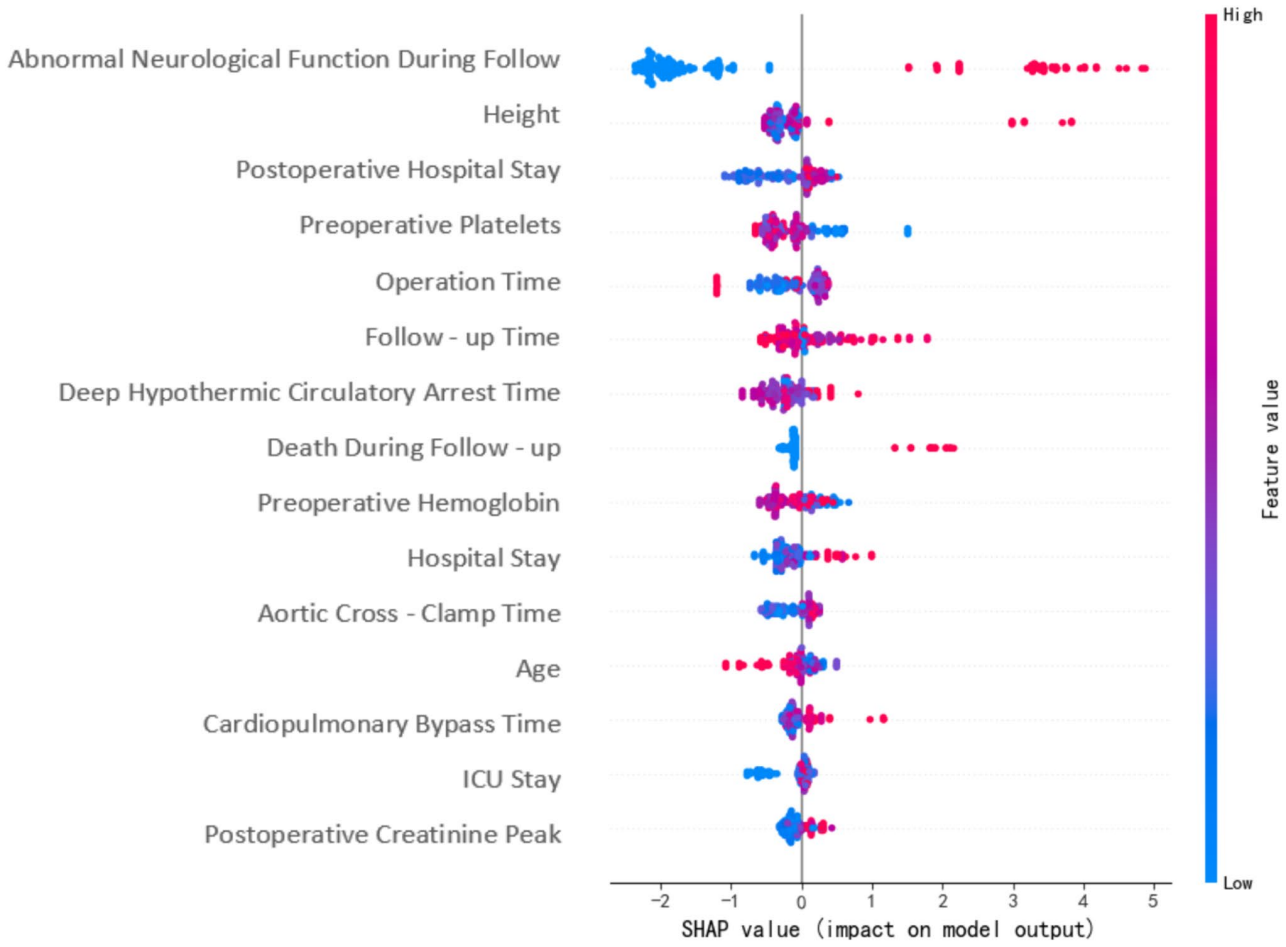
SHAP feature importance plot.

The SHAP method was used to analyze the importance of each feature in the XGBoost model. Figure 3 shows the results of the feature importance analysis, where more important features are distributed at the top and relatively less important ones at the bottom. Most features showed a positive or negative correlation with the prediction outcome. Abnormal levels (too high or too low) of preoperative platelets, preoperative hemoglobin, and deep hypothermic circulatory arrest duration increased the risk of postoperative ND.

**Figure 4:**
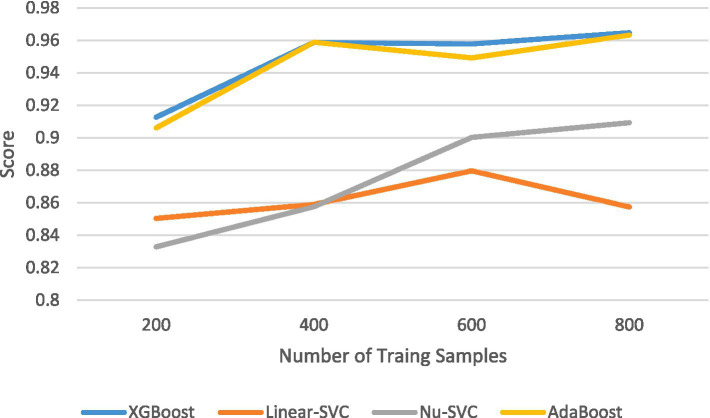
Learning curves.

The results of the comparison among the other four prediction models, calculated using internal validation data, are shown in Table 4. The ROC curves and corresponding AUC values are shown in Figure 2. The XGBoost model had an AUC of 0.966 (95% CI: 0.943–0.989), the Linear SVC model had an AUC of 0.859 (95% CI: 0.79–0.92), the Nu-SVC model had an AUC of 0.943 (95% CI: 0.89–0.98), and the AdaBoost model had an AUC of 0.957 (95% CI: 0.93–0.98). The learning curve of the model is shown in Figure 4, revealing how model accuracy changed as the number of training samples increased. Overall, the XGBoost model performed the best.

**Table 4:** Model comparison results.

Model	AUC	Accuracy	Recall	Precision	*F*_1_-score	Balanced accuracy
XGBoost	0.9661	90.97%	78.10%	88.42%	0.8220	0.8702
Linear-SVC	0.8593	87.92%	67.46%	87.05%	0.7506	0.8162
Nu-SVC	0.9433	84.41%	78.99%	69.95%	0.7377	0.8274
AdaBoost	0.9582	89.68%	73.48%	87.67%	0.7947	0.8471

#### 3.4.3 External validation

The external validation cohort included 375 patients, and the comparative features between the internal and external validation cohorts are shown in Table 5. The median age of patients in the external validation cohort was 41 years.

**Table 5:** Comparison of features between internal and external validation groups.

Characteristic	Internal validation (*n* = 853)	External validation (*n* = 375)
Total number	853	375
Age (year)	45 (51–60)	57 (37–65)
Gender, female *n* (%)	318 (37.28%)	280 (74.67%)
Weight (kg)	75 (65–81)	75 (69–90)
Height (m)	1.71 (1.62–1.77)	1.72 (1.67–1.76)
BMI (kg/m2)	26 (23–28)	26.02 (23.66–27.79)
Preoperative pericardial effusion *n* (%)	573 (67.17%)	243 (64.80%)
Smoking history *n* (%)	355 (41.62%)	183 (48.80%)
Hyperlipidemia *n* (%)	252 (29.54%)	57 (15.20%)
History of coronary heart disease *n* (%)	103 (12.08%)	37 (9.87%)
History of hypertension *n* (%)	641 (75.15%)	336 (89.60%)
History of diabetes mellitus *n* (%)	218 (25.56%)	79 (21.07%)
History of cerebrovascular disease *n* (%)	125 (14.65%)	52 (13.87%)
Preoperative renal dysfunction *n* (%)	46 (5.39%)	18 (4.80%)
Carotid artery stenosis or occlusion *n* (%)	188 (22.04%)	107 (28.53%)
History of cardiac surgery *n* (%)	107 (12.54%)	38 (10.13%)
Preoperative hypoxemia *n* (%)	53 (6.21%)	53 (14.13%)
Preoperative hemoglobin (g/L)	133 (121–148)	131 (124–137)
Preoperative platelets (*10^9^/L)	179 (146–217)	164 (129–225)
Preoperative creatinine (μmol/L)	83 (66–105)	92.7 (72–118.9)
Coronary artery involvement *n* (%)	384 (45.02%)	105 (28.00%)
Involvement of three branches of aortic arch *n* (%)	714 (83.70%)	284 (75.73%)
Operation time (hour)	7 (6–8)	7 (6–9)
Cardiopulmonary bypass time (min)	182 (144–218)	198 (174–211)
Aortic cross-clamp time (min)	98 (74–119)	105 (81–122)
Deep hypothermic circulatory arrest time (min)	22 (17–33)	22 (17–29)
Selective cerebral perfusion time (min)	22 (17–33)	22 (17–29)
Hospital stay (day)	15 (11–20)	17 (13–19)
ICU stay (hour)	72 (36–120)	48 (24–160)
Postoperative intubation time (hour)	31 (13–60)	32.5 (13–97)
Postoperative hospital stay (day)	12 (9–16)	14 (9–18)
Postoperative hypoxemia *n* (%)	402 (47.13%)	158 (42.13%)
Postoperative hemoglobin extremum (g/L)	79 (68–88)	81 (76–89)
Postoperative platelet extremum (*10^9^/L)	81 (54–102)	74 (53–129)
Postoperative creatinine peak (μmol/L)	125 (91–221)	122.9 (88.7–255.4)
Postoperative renal insufficiency *n* (%)	376 (44.08%)	142 (37.87%)
Postoperative low cardiac output syndrome *n* (%)	254 (29.78%)	50 (13.33%)
Reoperation *n* (%)	35 (4.10%)	0 (0.00%)
Abnormal neurological function during follow-up *n* (%)	215 (25.21%)	27 (7.20%)
Follow-up time (m)	36 (30–42)	12 (12–18)
Death during follow-up *n* (%)	79 (9.26%)	0 (0.00%)

**Figure 5:**
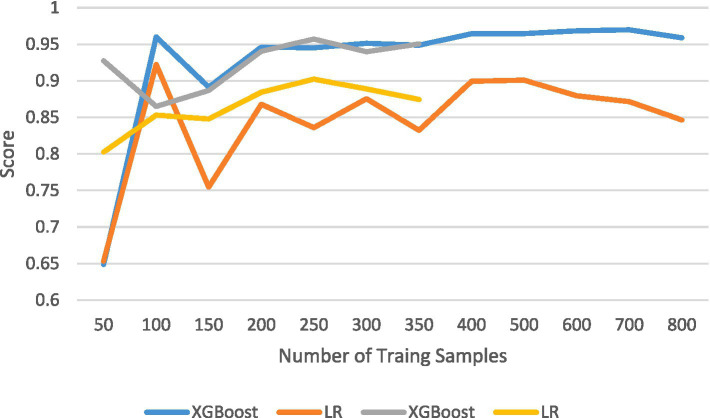
Comparison results between XGBoost and LR models.

The model was evaluated after model fitting using external validation data. The results showed that the XGBoost model achieved superior performance compared with the other three models, with an AUC of 0.951 (95% CI: 0.92–0.98). Among the other models, the Linear SVC model had an AUC of 0.870 (95% CI: 0.78–0.96), and the AdaBoost model had an AUC of 0.949 (95% CI: 0.92–0.98). The results of the comparison between the XGBoost and LR models are shown in Figure 5.

## 4 Discussion

### 4.1 Key findings

ND is one of the most common and destructive complications after ATAAD surgery, being associated with higher in-hospital mortality, postoperative renal and respiratory failure, longer ICU stay, longer ventilator support time, and lower long-term survival rate (5, 12). In this study, the incidence of postoperative ND in ATAAD patients was 27.78%, patients with ND experienced significantly delayed recovery, with longer ICU stay, longer ventilator use time, and significantly higher incidence of postoperative complications. Among them, the rates of postoperative hypoxemia, postoperative renal insufficiency, low cardiac output syndrome, and reoperation were significantly higher than those in the group without ND, suggesting a poor prognosis.

No prospective randomized data are currently available to guide the surgical strategy for ATAAD repair to prevent or reduce the risk of postoperative cerebrovascular events. Factors potentially influencing the development of ND after ATAAD surgery include the choice of arterial cannulation site, temperature management during cardiopulmonary bypass, cerebral protection strategy during hypothermic circulatory arrest, and the surgical approaches to the aortic arch and descending aorta during ATAAD surgery (7, 13).

Previous studies showed that the risk factors for ND include female sex, advanced age, previous cardiac surgery history, chronic obstructive pulmonary disease, diabetes history, prolonged cardiopulmonary bypass, and intraoperative transfusion of 2 or more units of red blood cells. This study found that the risk factors for postoperative ND in ATAAD patients were more complex. The analysis showed that sex, weight, height, history of cardiac surgery, history of cerebrovascular disease, smoking history, preoperative pericardial effusion, and preoperative renal insufficiency were independent risk factors affecting the occurrence of postoperative ND. Specifically, sex, weight, and height were risk factors for postoperative ND, suggesting that females and increased weight and height increased the risk of postoperative ND. History of cardiac surgery, history of cerebrovascular disease, preoperative pericardial effusion, and preoperative renal insufficiency were significant risk factors. Among them, preoperative renal insufficiency had the highest OR value, suggesting its closest association with postoperative ND; the history of cerebrovascular disease also showed a strong risk association, further confirming the important impact of preoperative cerebrovascular basic status on postoperative neurological function.

Early identification of patients at high risk of ND helps clinicians in the monitoring of patients and to take measures to prevent its occurrence. Many studies used risk factors or ML to construct prediction models for postoperative ND in ATAAD patients (14–16). Risk prediction is pivotal in cardiovascular disease research. LR is the most used traditional prediction model, but sometimes it cannot handle complex clinical data and cannot result in an ideal prediction model. In contrast, ML can handle complex clinical data, thus having more potential advantages (17). In this study, the four ML prediction models were compared through internal verification, and the XGBoost model performed better. The XGBoost algorithm easily adjusted parameters and handle non-linear features compared with other algorithms. It usually had higher sensitivity and specificity when overfitting was avoided. In most cases, XGBoost has higher prediction performance than other algorithms (18). The SHAP method explained the ML prediction model.

The SHAP method was used to analyze the importance of specific features in the prediction model. This method analyzes the impact of each variable on each patient, so the prediction model is interpretable (19). The SHAP method was effective in determining the importance of specific individual features. Most features were positively or negatively correlated with the prediction results, including excessively high or low preoperative platelets, preoperative hemoglobin, and deep hypothermic circulatory arrest time, which increased the risk of postoperative ND. After model fitting, the performance of the XGBoost model evaluated using external verification data was still superior to the other three models.

Finally, this study selected the optimal XGBoost model and compared it with the LR model. All features included in the LR model were also considered important features in the ML prediction model (20). However, the SHAP method also identified other features as important features, such as those that were not considered statistically significant in the LR model. This might lead to the LR model not including some relevant features, while the SHAP method did not exclude these features. According to the results of feature importance analysis, the impact of each variable on the results was assessed, guiding treatment decisions for patients and preventing the occurrence of postoperative ND.

### 4.2 Inverse probability of treatment weighting

To address the potential selection bias introduced by the single-center retrospective design of this study, the inverse probability of treatment weighting (IPTW) method was employed to balance confounding factors and verify the robustness of the model.

The standardized mean difference (SMD) was used to assess the distribution balance of 7 key confounding variables (including age, gender, BMI, preoperative pericardial effusion, deep hypothermic circulatory arrest time, etc.) between the exposure group and non-exposure group regarding arterial cannulation methods after IPTW weighting. As shown in Table 6, the weighted SMD values of all confounding variables were less than 0.1, indicating that IPTW weighting effectively eliminated the distribution differences in baseline characteristics and key intraoperative indicators between groups, and successfully controlled the interference of selection bias on effect estimation.

**Table 6:** SMD of key confounding variables after IPTW adjustment.

Confounding variable	SMD
Age	0.0117
Gender	0.0313
BMI (kg/m^2^)	0.0233
Preoperative pericardial effusion	0.0226
History of hypertension	0.0058
Deep hypothermic circulatory arrest time (min)	0.0155
Selective cerebral perfusion time (min)	0.0156

With postoperative ND as the outcome measure, the XGBoost prediction model was retrained using IPTW-weighted samples, and its discriminative performance was evaluated on the test set. Results showed that the AUC of the weighted model was 0.958. After controlling for selection bias, the model still maintained excellent predictive performance with robust effect estimation.

This outcome not only verifies the authenticity of the association between key variables (e.g., arterial cannulation methods) and the risk of postoperative ND, but also confirms the stability of the constructed XGBoost model under different bias control strategies, thereby providing a reliable efficacy basis for clinical application. Meanwhile, similar results were obtained when verifying other surgical protocols, including cardiopulmonary bypass (CPB). Therefore, it is concluded that the high-risk factors identified by the model and the recommended clinical strategies have a reliable inferential basis, which can be used to guide preoperative risk stratification and intraoperative decision optimization for patients with ATAAD.

### 4.3 Clinical applications and strategies

#### 4.3.1 Cannulation strategy: femoral vs. axillary artery cannulation

Reducing the occurrence of ND after ATAAD surgery remains a hot topic of debate in the cardiovascular field. The potential impact of femoral artery cannulation on the increase of the risk of postoperative ND is still controversial (21). Some studies showed that femoral artery cannulation has no effect on the incidence of ND (22, 23). In contrast, some other studies showed that femoral artery cannulation increases the risk of embolic events through the detachment of atherosclerotic, thrombotic, or other atherosclerotic embolic substances, leading to retrograde dissection tear and poor tissue perfusion (24, 25). There is increasing evidence that axillary artery cannulation is superior to femoral artery cannulation in combined aortic arch surgery (26).

In this study, the in-hospital mortality rate of patients with simple femoral artery cannulation was higher than that of patients with axillary artery cannulation. In our analysis, axillary artery cannulation was the main cannulation strategy for ATAAD patients. Axillary artery combined with femoral artery double cannulation was performed in the presence of lower limb arterial hypoperfusion to maintain sufficient tissue perfusion. After adjusting for relevant variables, patients with axillary artery cannulation had a significantly lower risk of postoperative ND compared with those with femoral artery cannulation. These findings provided a strong support in the prevention of postoperative ND by axillary artery cannulation, while femoral artery cannulation was associated with an increased risk of ND.

#### 4.3.2 Hypothermia and cerebral perfusion strategy

Existing studies and analyses showed that the incidence of postoperative ND is similar among all types of hypothermia strategies (including mild, moderate, and deep hypothermia) (27). However, deep hypothermia leads to the destruction of the postoperative coagulation mechanism, aggravates the damage to the circulatory system, and the transfusion of a large amount of blood is required during the operation to stabilize the body’s circulation, thereby prolonging the operation time, which is detrimental to the postoperative recovery of patients. Appropriately increasing the temperature during circulatory arrest and performing the operation under MHCA reduce the damage to various functions during the operation, result in faster cooling and rewarming, reduce the risk of ND, and contribute to the postoperative recovery of patients (28). According to the study by Samanidis et al. (29), retrograde cerebral perfusion is associated with a reduced risk of postoperative ND. However, the longer the cerebral perfusion time, the higher the risk of postoperative ND. Multiple retrospective studies confirmed the safety of MHCP combined with SACP (30, 31). Zierer et al. (23) studied 1,002 patients divided into unilateral cerebral perfusion group and bilateral cerebral perfusion group who underwent aortic arch surgery with mild hypothermia combined with antegrade cerebral perfusion. The incidence of postoperative ND was 3%, which was significantly lower than that in other retrospective studies. In this study, 7.65% of patients were not subjected to selective cerebral perfusion, which was not a risk factor for postoperative ND. No significant association was found between the body temperature during circulatory arrest and the occurrence of postoperative ND. However, the moderate hypothermic circulatory arrest time and selective cerebral perfusion time were longer than those in the group without ND, suggesting that surgical trauma and prolonged cerebral ischemia time might increase the risk of ND. The results of a German study on type A aortic dissection also showed the same result (12). Among 1,558 patients who underwent type A repair, 22.8% of patients were subjected to a cerebral protection strategy without auxiliary technology. Patients with a core temperature <15 °C were 2.6%, 15 °C to 20 °C were 59%, 21 °C to 25 °C were 27%, and 26 °C to 30 °C were 6%. The incidence of postoperative ND in the entire cohort was 13.4%, with no difference between different cerebral protection strategies (14.9% without cerebral protection, 14.1% with bilateral cerebral perfusion, 12.6% with unilateral cerebral perfusion), and the *p*-value was not significant. However, Svensson et al. (32) found that hypothermic circulatory arrest time exceeding 40 min significantly increases the risk of postoperative ND and mortality.

#### 4.3.3 Surgical strategies for the aortic arch

Some scholars believe that the aortic arch should not be repaired or only the proximal aortic arch should be repaired (i.e., hemiarch replacement) to reduce the risk of arch surgery (33). Postoperative results show that this method effectively saves patients’ lives, with relatively low postoperative mortality and complication rates. However, long-term follow-up shows that nearly 70% of patients still have dissecting lesions in the distal aorta due to the untreated distal aortic lesions, leading to the need for reoperation due to distal aortic dilatation or even aneurysm formation (34, 35). Studies showed that the reoperation rate within 10 years is 15–60% (36).

Recent progress in surgical technology, cerebral protection, and the application of new stent materials has led many Chinese and international centers to pursue a more active surgical approach, combining total aortic arch replacement with descending aorta stent implantation. Several studies show that this method eliminates the arch dissection and promotes the closure of the false lumen of the descending aorta. Long-term follow-up results confirm that the closure rate of the false lumen in the proximal descending aorta reaches 100%, thereby effectively reducing the incidence of long-term aortic complications and the risk of reoperation (37, 38). Sun et al. (39) reported the treatment of 291 cases of type A dissection with total arch replacement plus elephant trunk stent surgery, with a surgical mortality rate of only 3.1%, a spinal cord injury rate of 2.4%, a reoperation rate of 2.3%, and a false lumen closure rate of 94.2% in the proximal descending aorta. Although these results are encouraging, some scholars suggested that this method may increase the early risk after surgery. More surgical wounds, more complex surgical operations, and prolonged circulatory arrest time aggravate myocardial and cerebral ischemia and dysfunction of important organs, thereby increasing the postoperative mortality and complication rates (40). Kim et al. (41) reported that neurological complications are more common in ATAAD patients after total arch replacement, and the long-term postoperative survival rate is also lower. In addition, they also found that the reoperation rate is not related to the surgical method, and the mortality and complication rates of reoperation are lower. These evident contradictory results make the treatment strategy for acute aortic dissection arch lesions more confusing.

Island anastomosis refers to an arch surgical strategy that retains the proximal end of the supra-arch branches (innominate artery and/or left common carotid artery and/or left subclavian artery) and the arch vascular tissue and anastomoses them to the artificial blood vessel as an “island-shaped” vascular patch. Intimal repair was performed on the vascular island and the false lumen was closed at its base during the operation. This surgical method greatly simplifies the surgical operation of aortic arch replacement, reduces the separation and anastomosis operations of the supra-arch branch vessels, avoids damage to the surrounding tissues, and greatly reduces the circulatory arrest time. Studies showed that this method shortens the operation time, reduces postoperative complications, and provides better prognostic effects for patients (42). Considering that many ATAAD patients present complications with hereditary connective tissue diseases, including Marfan syndrome, the island anastomosis technique retains part of the autologous aortic wall, which is still at risk of aneurysmal dilatation in the long term. In addition, the part of the autologous aortic wall retained by the island anastomosis technique may be combined with residual dissection, which may compromise the long-term patency of the brachiocephalic vessels and increase the risk of ND (43).

No significant relationship was found between the mortality rate and the operation time of total aortic arch replacement, hemiarch replacement, and island anastomosis in the ND group and the control group. The cardiopulmonary bypass time and moderate hypothermic circulatory arrest time were positively correlated with the mortality rate, meaning that the longer the cardiopulmonary bypass time, the higher the mortality rate, and the longer the moderate hypothermic circulatory arrest time, the higher the mortality rate.

The moderate hypothermic circulatory arrest time of total aortic arch, hemiarch replacement, and island anastomosis in the ND group and the control group showed that different arch treatment methods did not have any direct relationship with the occurrence of ND. In terms of operation time, island anastomosis was longer than total aortic arch replacement and hemiarch replacement. In terms of cardiopulmonary bypass time, hemiarch replacement was shorter than island anastomosis and total aortic arch replacement. In terms of aortic cross-clamp time, hemiarch replacement was shorter than island anastomosis and total aortic arch replacement. In terms of moderate hypothermic circulatory arrest time, hemiarch replacement was shorter than island anastomosis and total aortic arch replacement. This suggested that aortic hemiarch replacement in the arch operation significantly shortened the circulatory arrest time, aortic cross-clamp time, and cardiopulmonary bypass time. Although island anastomosis reduced the difficulty of anastomosis of the arch branch vessels, it did not significantly improve the cardiopulmonary bypass time, aortic cross-clamp time, and circulatory arrest time, but instead increased the operation time, which is considered as highly related to intraoperative anastomotic bleeding.

## 5 Conclusion

Leveraging clinical data from 1,228 patients diagnosed with Acute type A Aortic Dissection (ATAAD), the study developed a risk prediction model for postoperative ND following ATAAD surgery. The model construction integrated SHAP-based feature selection and the XGBoost algorithm. Key findings are presented as follows:

1. Fifteen core features identified through screening—including deep hypothermic circulatory arrest duration, preoperative platelet count, and operative time—demonstrated efficacy in predicting the risk of postoperative ND.
2. The XGBoost model achieved the optimal predictive performance, with AUC of 0.966 in internal validation and 0.951 in external validation. It outperformed the conventional Logistic Regression (LR) model significantly, while also exhibiting favorable calibration and high clinical applicability.
3. Preoperative renal dysfunction, a history of cerebrovascular disease, prolonged operative duration, and cardiopulmonary bypass were confirmed as critical risk factors for post-operative ND in ATAAD patients.

In conclusion, this model may assist clinicians identifying patients at high risk of ND, providing evidence to inform individualized treatment decision-making, offering clinical guidance, and enabling timely intervention. Nevertheless, its clinical implementation necessitates further validation through multi-center prospective studies.

### 5.1 Limitation

This research is subjected to inherent limitations since it is a single-center retrospective study. Potential unmeasured treatment selection bias or confounding factors should be considered when formulating surgical strategies and the findings should be interpreted accordingly.

The definition of postoperative ND in this study was based solely on clinical manifestations, without the incorporation of neuroimaging data. Consequently, some patients with cerebral hypoperfusion might have been underdiagnosed. Additionally, intraoperative cerebral oxygen monitoring data were absent from the surgical records, which might also be relevant to the intraoperative cerebral perfusion status.

Other limitations of this study include the lack of computed tomography (CT) data and information on key variables, including the specific location of the primary aortic tear. In this study, the operative duration for island anastomosis of the aortic arch was longer than that for total arch replacement and hemi-arch replacement. Although the circulatory arrest time for island anastomosis was similar to that for total arch replacement, follow-up assessment revealed a residual dissection in the arch branch vessel of some patients, which contributed to the development of neurological complications during the follow-up period. This observation might be attributed to the failure of island anastomosis to fully resolve residual dissection in the arch branch vessels. Furthermore, study records indicated that 30% of patients underwent ascending aortic replacement; however, some of these patients probably received hemi-aortic arch replacement, which was incorrectly documented as ascending aortic replacement. The database also lacked records specifying whether axillary artery cannulation was performed through a side graft or direct cannulation of the axillary artery.

## Data Availability

The original contributions presented in the study are included in the article/Supplementary material, further inquiries can be directed to the corresponding authors.
